# Effects of Short- and Long-Term Soy Protein Feeding on Hepatic Cytochrome P450 Expression in Obese Nonalcoholic Fatty Liver Disease Rat Model

**DOI:** 10.3389/fnut.2021.699620

**Published:** 2021-06-28

**Authors:** Melisa Kozaczek, Walter Bottje, Diyana Albataineh, Reza Hakkak

**Affiliations:** ^1^Department of Dietetics and Nutrition, University of Arkansas for Medical Sciences, Little Rock, AR, United States; ^2^Department of Poultry Science and The Center of Excellence for Poultry Science, University of Arkansas, Fayetteville, AR, United States; ^3^Arkansas Children's Research Institute, Little Rock, AR, United States; ^4^Department of Pediatrics, University of Arkansas for Medical Sciences, Arkansas Children's Hospital, Little Rock, AR, United States

**Keywords:** soy protein isolate, liver steatosis, obesity, cytochrome P450, NAFLD

## Abstract

Obesity can lead to chronic health complications such as nonalcoholic fatty liver disease (NAFLD). NAFLD is characterized by lipid aggregation in the hepatocytes and inflammation of the liver tissue as a consequence that can contribute to the development of cirrhosis and hepatocellular carcinoma (HCC). Previously, we reported that feeding obese Zucker rats with soy protein isolate (SPI) can reduce liver steatosis when compared with a casein (CAS) diet as a control. However, the effects of SPI on cytochrome P450 (CYP) in an obese rat model are less known. In addition, there is a lack of information concerning the consumption of soy protein in adolescents and its effect in reducing the early onset of NAFLD in this group. Our main goal was to understand if the SPI diet had any impact on the hepatic CYP gene expression when compared with the CAS diet. For this purpose, we used the transcriptomic data obtained in a previous study in which liver samples were collected from obese rats after short-term (eight-week) and long-term (16-week) feeding of SPI (*n* = 8 per group). To analyze this RNAseq data, we used Ingenuity Pathway Analysis (IPA) software. Comparing short- vs long-term feeding revealed an increase in the number of downregulated CYP genes from three at 8 weeks of SPI diet to five at 16 weeks of the same diet (*P* ≤ 0.05). On the other hand, upregulated CYP gene numbers showed a small increase in the long-term SPI diet compared to the short-term SPI diet, from 14 genes at 8 weeks to 17 genes at 16 weeks (*P* ≤ 0.05). The observed changes may have an important role in the attenuation of liver steatosis.

## Introduction

Obesity is a multifactorial disease that can result from an imbalance between high energy intake and low energy expenditure due to reduced physical activity (1). Obesity can increase the clinical risks for developing other health conditions, such as cardiovascular disease, insulin resistance, type 2 diabetes, and nonalcoholic fatty liver disease (NAFLD) (2, 3). NAFLD is one of the most frequent and widespread causes of liver disease in the United States and is commonly complementary in patients with other metabolic disorders (4–6). The prevalence of NAFLD ranges from 10 to 30% worldwide and affects 30% of the population in the United States, thus becoming an international health epidemic (4, 7). The pathology of NAFLD represents a spectrum of stages from mild steatosis to nonalcoholic steatohepatitis (NASH), often accompanied by inflammation, and fibrosis or cirrhosis that can lead to hepatocellular carcinoma (HCC) if left untreated (4, 8, 9). NAFLD is a multisystem disease that develops from insulin resistance, caused by an excessive accumulation of lipids in hepatocytes, followed by the alteration of oxidative stress, the accumulation of endotoxins, and the accumulation of inflammatory cytokines in the liver (10, 11).

The liver is the primary organ in the human body that functions to metabolize and detoxify xenobiotic molecules, including exogenous toxins and drugs (12). The cytochrome P450 (CYP) superfamily is one of the main drug-metabolizing families of heme-containing enzymes, playing a key role in protecting the organism from toxic compounds, both endogenous and exogenous (13, 14). The CYP superfamily receives its name from the fact that they exhibit a maximum absorbance at the wavelength of 450 nm when bound to carbon monoxide (15). This conspicuous set of proteins found in all biological kingdoms comprises oxidases, reductases, and hydrolases: most of them are part of Phase I drug and toxin metabolism that serves in making the target compounds more hydrophilic and more easily excreted either in the bile or in the urine (12, 15). In NAFLD, there are altered functions and storage of adipose tissues, upregulation of proinflammatory biomarkers, production of reactive oxygen species (ROS), and changes in gene expression (16). Major families of CYP proteins are shown to be differentially expressed in the liver throughout the progression of NAFLD (17, 18). In addition, the dietary composition can alter the expression and activity of many CYP proteins, which can, in turn, influence the drug metabolism and disease prevalence (19, 20). In this regard, soy proteins have been extensively studied, mostly due to their correlation with health benefits such as the prevention of chronic diseases and some types of cancer (21, 22).

Soybeans are the main source of protein in Asian diets, vegetarians, and soy infant formula (23). Multiple studies concerning the effects of soy diet in rat models have been performed; however, to our knowledge, most of this research has been carried out on nonobese rat lines (24–26), and very little is known concerning the effects of soy protein isolate (SPI) diet in an obese rat model and its preventive effects against NAFLD (27). There is also a need for more information pertaining to the inclusion of soy proteins in the diets of infants and adolescents (28). Thus, this study focuses on the impact an SPI diet has in obese juveniles (15 weeks old, 8 weeks on diet, short-term SPI diet) and adult rats (23 weeks old, 16 weeks on diet, long-term SPI diet). We can extrapolate both the short- and long-term results to adolescents and adults, respectively. Recently, we reported that SPI had a protective effect against liver steatosis (29) and proposed that SPI reduces inflammation and enhances the lipid transport out of the liver in obese Zucker rats (30, 31).

There are very limited data on the effects of soy protein feeding in the NAFLD model on the expression of CYP450; therefore, the main objectives of this study were to investigate the effects of short- and long-term SPI on hepatic CYP450 expression.

## Materials and Methods

### Ethics Statement

This study used the methods described in the studies by Hakkak et al. (29) and Kozaczek et al. (32), and the description of the methods partly reproduces their wording (29, 32). The protocol for this study (code number 3242) was approved by the Institutional Animal Care and Use Committee at the University of Arkansas for Medical Sciences (IACUC) on December 6, 2011.

### Experimental Design

Six-week-old male Zucker rats (*n* = 8–9 per group) were purchased from Envigo (Indianapolis, IN). After 1 week of acclimation, 7-week-old rats were randomly assigned to diets containing either SPI casein (CAS, control) as the main protein source for 8 and 16 weeks. Rats were weighed two times per week and had *ad libitum* access to feeding and water. After 8 weeks of diet, when the rats were 15 weeks old, half of the rats in the SPI group and the CAS group were sacrificed. In this stage, rats were juveniles and the results can be extrapolated to adolescents. The remaining obese Zucker rats continue to be on their respective diets (either SPI or CAS) for another 8 weeks to double the amount of time on experimental feeding, making a total of 16 weeks of diet. After 16 weeks on experimental diets, when the rats were 23 weeks old, all the rats were sacrificed. Rats were anesthetized with carbon dioxide and euthanized by decapitation at the end of each experiment, at 8 (15-week-old rats) and 16 weeks (23-week-old rats) of SPI diet. Blood and liver samples were collected. Liver tissues were immediately flash-frozen with liquid nitrogen and stored at −80°C. Envigo prepared both diets, and the composition of both diets is described in Table 1.

**Table 1:** Diet composition (33).

**Ingredients**	**Casein (g/kg)^a^**	**Soy protein (g/kg)^a^**
Casein	200	–
Soy Protein Isolate^b^	–	202
l-Cystine	3	1.3
l-Methionine	–	2.5
l-Tryptophan	–	0.4
l-Threonine	–	0.3
Corn starch	397.5	409
Maltodextrin	132	132
Sucrose	100	108
Corn oil^c^	70	63
Cellulose	50	50
AIN-93 G Mineral mix	35	35
AIN-93 G Vitamin mix	10	10
Choline bitartrate	2.5	2.5
TBHQ, antioxidant	0.014	0.014

a *Gram of ingredient/kg of diet*.

b *Soy protein isolate contained bioactive components of high isoflavones, which include 3.24 mg total isoflavones/g protein (1.88 aglycone equivalents/g protein)*.

c *The amount of corn oil was adjusted in the soy protein diet to account for the fat contribution from soy protein*.

### Transcriptomics/CYP Analysis

The methods for RNA extraction from the liver samples and the pipeline followed to generate the RNAseq data are described in Kozaczek et al. (32). More than 1,200 transcripts were differentially expressed (>1.3-fold difference and *P* < 0.05) and later evaluated with Ingenuity Pathway Analysis program (IPA, Qiagen, CA) to help in the analysis and understanding of the global gene expression data. To illustrate the differentially expressed genes in relative values, we used the scientific graphing software Graph Pad Prism 8.4.3 (La Jolla, CA) and Student's *t*-test. Differences were considered significant at *P* < 0.05. Transcriptomic data are available in the Gene Expression Omnibus database (GEO accession number GSE158553). The transcriptomic analysis is based on the statistical analysis obtained using the IPA application to compare the gene expression of CYP450 in results of the SPI diet with that in the results of the CAS control diet. IPA software analysis algorithm generates the predictions of activation or inhibition of upstream regulator molecules and downstream functions calculating two statistical measures. These two statistical measures are based on both the scientific literature stored in the Qiagen knowledge database and the activation state of the molecules in our datasets. These statistical measures are the activation *Z*-score and *p*-value of overlap. If the expression of the molecules in our datasets is mainly consistent with the gene expression reported in the stored bibliography database, then IPA generates a *Z*-score to most likely predict the activation/inhibition state of each molecule. *Z*-scores over 2.0 represent the activation, and *Z*-scores below −2.0 represent the inhibition. The *p*-value of overlap measures if the overlap between molecules in our dataset and known upstream regulators is statistically significant and is calculated using Fisher's exact test. Significance is attributed to overlap *p* < 0.05. Any molecule with the ability to affect the expression of other molecules is considered an upstream regulator. Master regulators are the molecules that regulate other transcriptional regulators. Further, it is important to specify that each set of data, 8 and 16 weeks of diet, has already integrated the comparison between the SPI and the CAS diet results. In other words, the differential gene expression and the predicted activation states of each molecule are already calculated against the CAS diet results. Furthermore, every prediction in one direction (upregulated or downregulated) in the SPI diet dataset has the opposite direction in the CAS diet. For example, if a gene or function is upregulated or predicted to be activated in the SPI diet, it is downregulated or predicted to be inhibited in the CAS diet and vice versa. All the fold differences in expression are relative values, showing gene expression with the SPI diet compared with expression with the CAS diet. More detailed information about the algorithms used by IPA application is explained in this study by Krämer et al. (34).

## Results

The average initial body weight (BW) at the beginning of the experiment (7-week-old rats) for the CAS group expressed in grams (g) was 199 ± 17, whereas it was 202 ± 17 for the SPI group. At 8 weeks of diet, the final BW for the CAS group was 548 ± 66, whereas the BW for CAS at 16 weeks of diet was 658 ± 62. The final BW for the SPI group after 8 weeks of diet was 605 ± 55, whereas the final BW for the SPI group at 16 weeks of diet was 735 ± 25. The average liver weight for the CAS group at 8 weeks of diet was 25.7 ± 1.5 and that for the CAS group at 16 weeks of diet was 30.9 ± 4.9.

Liver weight for the SPI group at 8 weeks of diet was 20.9 ± 3.9, and the liver weight for the SPI group at 16 weeks of diet was 22.0 ± 2.1. SPI-fed rats had substantially lower liver weight than CAS-fed rats after both 8 and 16 weeks of SPI feeding (29). Steatosis was calculated as a score of 1 to 4 based on the observed lipid accumulation within hepatocytes: 1) <25%, 2) 25–50%, 3) 50–75%, and 4) >75%. After 8 weeks of experiment (15-week-old rats, the short-term SPI feeding), the SPI-fed rats had a liver steatosis score of 2.10 ± 1.0, whereas the CAS-fed rats had a steatosis score of 3.20 ± 0.5 (*P* < 0.05). After 16 weeks of experiment (23-week-old rats, the long-term SPI feeding), SPI-fed rats had a steatosis score of 1.70 ± 0.7, which was significantly lower (*P* < 0.001) than the CAS-fed rats with a steatosis score of 3.4 ± 0.9. Serum inflammatory biomarkers ALT and AST levels were published in the study by Hakkak et al. (29).

Previously, we reported the results of feeding SPI-based diets in both male (29) and female (35, 36) Zucker rats, where the SPI-fed rats (females and males) were equally protected against liver steatosis compared to the CAS-fed rats. In addition, we reported the effects of SPI feeding for eight and 16 weeks on targeted liver gene expression obtained by RNAseq and validation using the real-time PCR (RT-PCR) in obese Zucker rats (32, 37). In this report, we show that feeding SPI for short term and long term affected the expression of CYP genes in the livers of Zucker rats compared to those fed with CAS.

**Figure 1:**
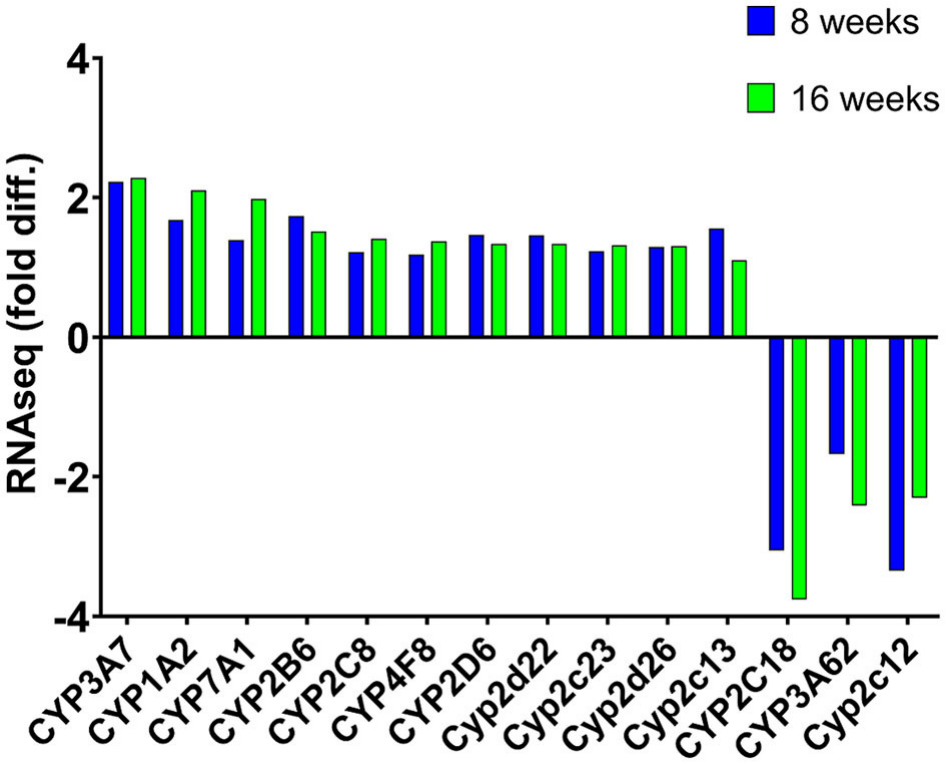
Mean expression (fold difference) of differentially expressed cytochrome P450 (CYP) genes (listed in IPA) that were observed to be expressed at both 8 and 16 weeks in rats fed with the SPI diet compared to those fed with the CAS diet. Positive values indicate upregulation, whereas negative values represent downregulation of these genes. Values represent the mean of 8–10 observations and bars with an asterisk (*) were significant (*P* < 0.05). Cyp2c13 was significant after 8 weeks of soy feeding (*p* = *4.58* × *10*^−05^) but not after 16 weeks of soy feeding (*p* = 0.24).

We compared which CYP genes were upregulated and downregulated at 8 and 16 weeks of SPI diet compared to the CAS diet (Figure 1). The statistics measurements used in this study were the comparison of *p*-values, relative fold changes, and *Z*-scores generated through analysis with IPA software. All the results presented in this study were compared and are relative to the expression levels in the CAS control diet results. Most of the CYP genes belong to one of the following main families of CYP, CYP1, CYP2, and CYP3, with some exceptions belonging to CYP4 and CYP5 for the downregulated CYP genes (Table 2), and CYP7 subfamily in the upregulated group (Table 3). After 8 weeks of SPI feeding, only three CYP genes were downregulated: Two genes belong to the CYP2C subfamily and the other gene belongs to the CYP3A subfamily. By the 16th week, five CYP genes were downregulated in the SPI-fed group (Table 2). On the other hand, the number of upregulated CYP genes did not change significantly between 8 and 16 weeks of the SPI diet. Rats fed with SPI diet for 8 weeks presented 14 upregulated CYP genes (Table 3), whereas the rats fed with SPI for 16 weeks presented 17 upregulated CYP genes.

**Table 2:** Downregulated CYP expression comparison between soy protein isolate (SPI) vs. casein (CAS) feeding at 8 and 16 weeks of diet.

**8 weeks**		**16 weeks**	
**Gene**	***p*-value**	**Gene**	***p*-value**
CYP2C12	*1.76 × 10^−17^*	CYP2C18	*9.92 × 10^−14^*
CYP2C18	*1.12 × 10^−10^*	CYP3A62	*6.23 × 10^−13^*
CYP3A62	*1.75 × 10^−04^*	CYP2C12	*6.06 × 10^−08^*
		CYP2C70	*1.15 × 10^−06^*
		CYP4A14	*4.89 × 10^−02^*

*All p-values were statistically significant (p ≤ 0.05)*.

**Table 3:** Upregulated CYP expression comparison between SPI versus CAS feeding at 8 and 16 weeks of diet.

**8 weeks**		**16 weeks**	
**Gene**	***p*-value**	**Gene**	***p*-value**
CYP3A7	*5.48 × 10^−07^*	CYP1A2	*5.97 × 10^−12^*
CYP2C13	*4.58 × 10^−05^*	CYP2B9	*3.92 × 10^−08^*
CYP1A2	*5.40 × 10^−05^*	CYP7B1	*1.07 × 10^−06^*
CYP2D26	*5.17 × 10^−04^*	CYP3A7	*1.66 × 10^−06^*
CYP2D6	*1.31 × 10^−03^*	CYP7A1	*4.02 × 10^−06^*
CYP2D22	*1.91 × 10^−03^*	CYP4F8	*8.98 × 10^−05^*
CYP2B6	*2.05 × 10^−03^*	CYP2C8	*4.80 × 10^−04^*
CYP2B9	*3.12 × 10^−03^*	CYP2C23	*1.08 × 10^−03^*
CYP2D1	*9.52 × 10^−03^*	CYP2D26	*1.43 × 10^−03^*
CYP2C19	*1.17 × 10^−02^*	CYP2D1	*9.10 × 10^−03^*
CYP2C23	*1.25 × 10^−02^*	CYP3A25	*1.31 × 10^−02^*
CYP4F8	*5.00 × 10^−02^*	CYP2D22	*1.52 × 10^−02^*
CYP2C8	5.39 × 10^−02^	CYP27A1	*1.52 × 10^−02^*
CYP7A1	5.98 × 10^−02^	CYP2C9	*1.65 × 10^−02^*
		CYP2B6	*1.80 × 10^−02^*
		CYP2A12	*2.04 × 10^−02^*
		CYP2D6	*3.85 × 10^−02^*

*Values in italic font were statistically significant. Values not italicized were borderline significant (p ≤ 0.05)*.

**Figure 2:**
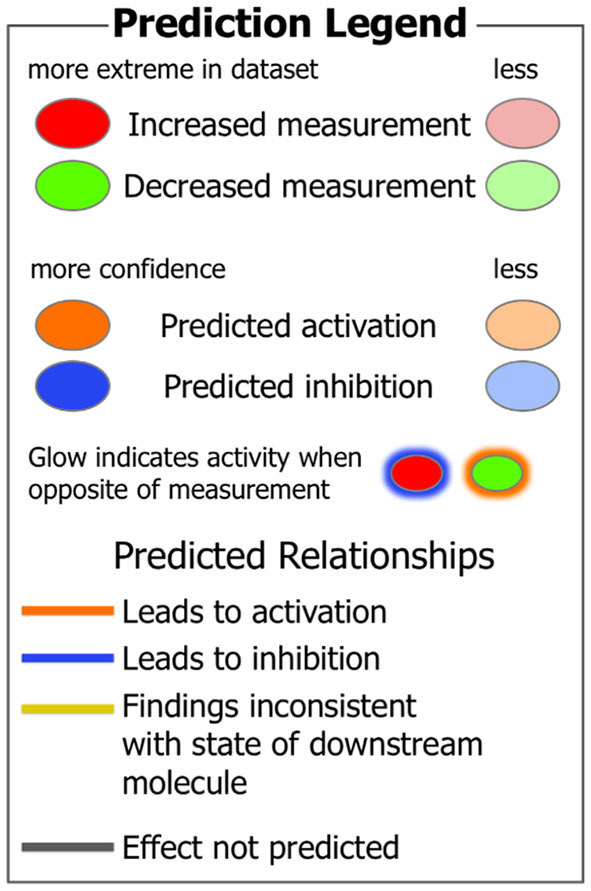
IPA Prediction legend to help in the understanding of regulator networks.

**Figure 3:**
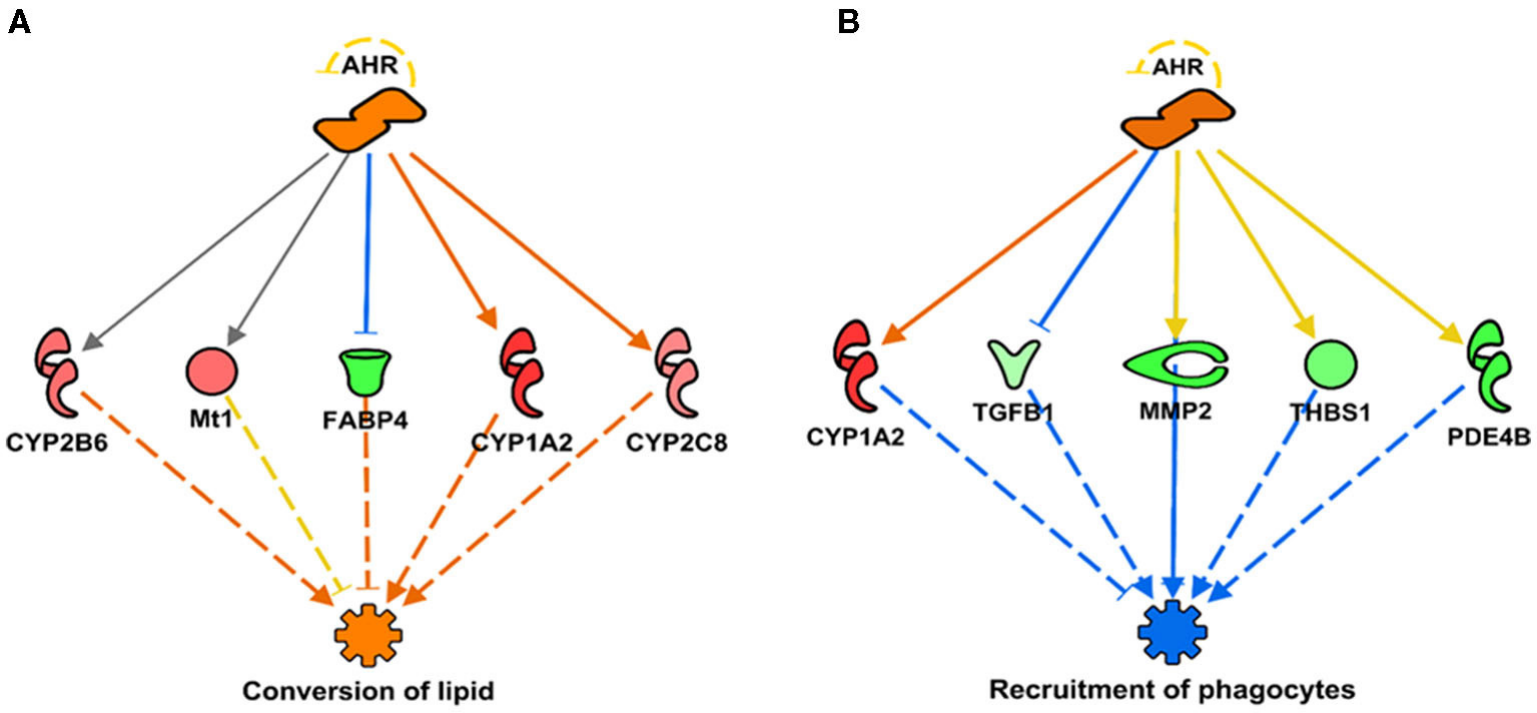
Regulator networks illustrating the role of aryl hydrocarbon receptor (AHR) as the master regulator in a component of lipid metabolism and inflammatory response. **(A)** Conversion of lipid (*Z*-score = 2.09, *p* = *2.77* × *10*^−08^), predicted to be activated in SPI diet; **(B)** recruitment of phagocytes (*Z*-score = –2.31, *p* = *2.10* × *10*^−05^), predicted to be inhibited. Both networks have in common the upregulation of CYP1A2 as indirect result of AHR activation (activation *Z*-score = 2.15, *p* = *4.20* × *10*^−11^). Genes in pink or red were upregulated in the SPI-fed rats, whereas those in green were downregulated in SPI- vs. CAS-fed rats. Gene names and fold differential expression in the regulatory networks are CYP1A2 (Cytochrome P450 Family 1 Subfamily A Member 2, 2.11), FABP4 (Fatty Acid Binding Protein 4, −1.91), CYP2B6 (Cytochrome P450 Family 2 Subfamily B Member 6, 1.51), Mt1 (Metallothionein 1, 1.52), CYP2C8 (Cytochrome P450 Family 2 Subfamily C Member 8, 1.41), TGFB1 (transforming growth factor beta 1, **–**1.33), MMP2 (matrix metallopeptidase 2, **–**1.96), PDE4B (phosphodiesterase 4B, **–**1.93), and THBS1 (thrombospondin 1, **–**1.63).

In addition, we report a predicted activation of aryl hydrocarbon receptor (AHR) linked to the subsequent activation or upregulation of various CYP genes (Figures 2, 3). This prediction of AHR activation was obtained with the “regulator effect” analysis function on IPA. The regulator effect algorithm connects upstream regulators with downstream molecules and downstream functional outcomes. It can provide an understanding of how the activation or inhibition of an upstream regulator could be affecting the expression of other molecules and important functions in our dataset. The regulator effect generates directional networks based on our input data and a wide range of scientific literature stored on the IPA database, thus generating a hypothesis on how specific phenotypes or functions could be regulated in our dataset by activated or inhibited upstream regulators (genes). The generated networks are displayed in a hierarchical manner, with a master regulator at the top (AHR in this case), dataset molecules in the middle, and a phenotype or function at the bottom (in Figure 3, the functions are the conversion of lipid and the recruitment of phagocytes). The master regulator would ultimately have an activating or inhibiting effect over the function at the bottom through the downstream molecules that are present in the dataset. Figure 2 shows a prediction legend for the interpretation of regulator networks. Red molecules are upregulated, whereas green molecules are downregulated. Orange molecules are predicted to be activated and blue molecules are predicted to be inhibited as a result of the interaction with the master regulator. Uninterrupted arrows indicate the direct molecule–molecule interactions, while dashed arrows indicate that between those two molecules are intermediary molecules or steps. Gray arrows are displayed when there are not enough reports on the interactions of genes in the scientific literature stored in the IPA knowledge database; therefore, IPA software does not predict activation or inhibition (Figure 3A). Figure 3 shows how AHR activation was predicted to indirectly activate or inhibit two main metabolic functions under SPI feeding: conversion of lipid (lipid metabolism)—predicted to be activated (Figure 3A), and recruitment of phagocytes (inflammatory response)—predicted to be inhibited (Figure 3B).

## Discussion

The first signs of NAFLD are a detrimental aggregation of lipids within the liver cells, leading to insulin resistance and inflammation-causing steatosis that can later develop into fibrosis and NASH and finally lead to cirrhosis and HCC (38, 39). Studies have shown a dysregulation in CYP proteins during the progression of NAFLD (40). CYP protein families are found in high concentrations in the liver and are notably responsible for drug and exogenous substrate metabolism, detoxification of environmental chemicals, bioactivation of carcinogens, and the biosynthesis of endogenous substrates (41, 42). In addition, they play significant roles in metabolizing noncellular materials, cholesterol, vitamin D, arachidonic and retinoic acids, eicosanoids, and the biosynthesis of bile acids and steroids (43). CYP proteins function to metabolize fatty acids by hydroxylating terminal carbons in the fatty acid chain (44). During the process of metabolizing fatty acids, the hydroxylated fatty acids are converted into dicarboxylic acids that are then metabolized by the beta-oxidation system in the peroxisomes of liver cells (45). This system converts the dicarboxylic acid to the shorter fatty acids that are transported to the mitochondria for the final oxidation step. The energy supplied by this oxidation is used for the lipid synthesis or are transported to the peripheral tissues when the energy production is low (46). In the liver, the hydroxylation of fatty acids and their metabolism are used as a mechanism for energy production, lipogenesis, and structural lipid synthesis (46). In the normal human liver, the factors that influence the expression and regulation of CYP proteins are imbalances of endocrine hormones, poor diet, genetic polymorphism, and environmental factors (47). Chronic liver disease is a dominant force that impairs CYP drug metabolism function in the liver cell (47). Altered CYP protein regulation and expression are positively correlated with the severity of liver disease, most notably in NAFLD (47). Decreased CYP protein activity directly affects the metabolism of the therapeutic drugs that can have a negative impact on cell toxicity (47). In contrast, increased activity of CYP enzymes can lead to an excessive metabolism of cellular substrates that can cause an increase of ROS in the cell (47). Multiple individual CYP proteins have been associated with particular disorders (48). For example, overexpression of CYP7A1 seems to protect against atherosclerosis by reducing the accumulation of visceral fat among other factors (48), whereas downregulation of CYP1A2 has been correlated with the progression of HCC (49). CYP1A2 and CYP7A1 were highly upregulated in this study at both eight and 16 weeks of SPI feeding (Figure 1 and Table 3), showing an increase in their expression in the long-term feeding.

CYP1, CYP2, and CYP3 protein family members dominantly metabolize an estimated 75% of all hepatic drugs in humans and animals (50, 51). In addition, animal studies suggest that an accumulation of lipids in hepatocytes can impair CYP protein function (52) as found in patients with NAFLD that present with a downregulation of nuclear receptors during the transcriptional regulation of Phase I and Phase II drug-metabolizing enzymes (53). In addition, it has been established in recent years that CYP epoxygenases, the enzymes catalyzing the oxidation of polyunsaturated fatty acids (PUFA) to PUFA epoxides, play an essential role in the regulation of inflammation through the production of these reactions, which can act as lipid mediators (54). Various CYP subfamilies have been categorized as CYP epoxygenases, including CYP1A, CYP2B, CYP2C, CYP2E, and CYP2J subfamilies. In humans, CYP2C8, CYP2C9, CYP2C19, and CYP2J2 isoforms have been recognized as CYP epoxygenases converting arachidonic acid into different types of PUFA epoxides (55). In this study, CYP2C8, CYP2C9, and CYP2C19 (Table 3) were upregulated at both 8 and 16 weeks in SPI-fed obese rats. Validation by RT-PCR of CYP2C12 expression (16 weeks, *p* = *6.06* × *10*^−08^) was previously reported in the study of Kozaczek et al. (32). CYP7A1, CYP1A2, CYP2B6, CYP2C19, CYP2D6, CYP2E1, and CYP4F8 mRNA enzymatic activity and protein expression are reported to significantly decrease in response to hepatic inflammation, steatosis, and the progression of NAFLD in the liver (56–58), as shown in this study in the CAS control group presenting more liver inflammation than SPI group results.

CYP4 family of enzymes has been reported to increase its expression after feeding SPI vs. CAS (59). The ability of some CYP4F subfamily members to break down and metabolize pro- and anti-inflammatory leukotrienes illustrates that this subfamily may have an important role in the activation and resolution stages of inflammatory responses in the body (60). CYP4F8 utilizes prostaglandin E (PGE) synthase isomerization of prostaglandin endoperoxides (PGH_1_) and (PGH_2_) to metabolize PGH_1_ and PGH_2_ to 19-OH PGE1 and 19-OH PGE 2. PGE is known to have a large effect on cytokine synthesis and therefore can play an important role in the resolution phase of inflammation (61). CYP4F proteins are noted to play a role in lipid homeostasis by affecting lipid accumulation during the progression from steatosis to steatohepatitis through the stimulation of inflammatory immune cells (61). These previous reports concur with our results. The fact that CYP4F8 was strongly upregulated in the present study at both 8 and 16 weeks of SPI-fed rats (Table 3) and, consequently, downregulated in CAS-fed rats, may indicate an important role that CYP4F8 could be playing in reducing liver inflammation, which is observed to be decreased in the livers of SPI-fed rats. In addition, CYP4A14 upregulation was shown in the livers of patients with NAFLD and in several murine NAFLD models, and a deficiency or inhibition of CYP4A14 has been established to decrease hepatic inflammation and fibrosis (62). Our results corroborate this statement since CYP4A14 was downregulated after 16 weeks of SPI diet (Table 2), and its downregulation could be directly related to the observed amelioration of liver steatosis in obese Zucker rats.

Aryl hydrocarbon receptor is a ligand-activated helix-loop-helix cytosolic transcription factor involved in the control of main functions such as cell proliferation, differentiation, immune system, and apoptosis (Figure 2) (63, 64). AHR is a known regulator of xenobiotic metabolism, along with pregnane-X-receptor (PXR), constitutive androstane receptor (CAR), and peroxisome proliferator-activated receptor alpha (PPARα) (65). Upon ligand binding, the translocation of AHR into the nucleus occurs where it regulates the expression of many target genes, including xenobiotic-metabolizing enzymes such as CYP1A1 and CYP1A2 (64, 66). AHR has been extensively studied in its relationship with CYP1A1 and cancer prevention (67–69). Studies have shown that flavonoids and other natural compounds in the diet are mostly AHR agonists but that there are also studies that classify them as AHR antagonists (68). There is also evidence that the expression of CYP1A1 has protective effects against liver steatosis (70). Dietary flavonoids have also shown to have a protective role in gastrointestinal inflammation, probably acting on the gut microbiota and regulating interactions with AHR and estrogen receptors (71). Through the direct upregulation of CYP1A2 observed in this study and the indirect (prediction of) inhibition of important functions of the inflammatory response, such as the recruitment of phagocytes (Figure 3B), the activation of AHR by SPI diet could be contributing to the attenuation of inflammation in the liver tissue. Previously, we discussed how the SPI diet could be reducing liver steatosis by enhancing the lipid metabolism of the liver and inhibiting the inflammatory response in the liver tissue (37). The predicted activation of AHR by SPI feeding in this study could be part of the explanation for the observed amelioration of liver steatosis in SPI-fed obese rats compared with CAS-diet-fed rats.

We consider it is important to highlight that CYP protein isoforms and their expression can vary between rodent models and humans, in addition to the utilization of different animal models and experimental designs (72, 73). However, studies have shown a correlation between obesity and NAFLD with the upregulation or downregulation of particular CYP families that could be triggered by inflammatory mediators such as interleukin 1β (IL-1β) and IL-6 (74, 75). Serum levels of these inflammatory mediators are usually increased in obese patients with NAFLD and the obese Zucker rat model, as reported in the studies by Hakkak et al. (29) and Kozaczek et al. (30). We also reported how feeding SPI reduces the expression of inflammation-related genes, such as IL-33 and IL-1B, that could be helping in the general reduction of liver steatosis observed with the SPI diet (31). In humans, studies have described a reduction in the expression of CYP3A family related to liver inflammation (76), and a study by Woolsey et al. (77) reported a reduction of CYP3A family in humans and a murine model for NAFLD. In 2017, Maximos et al. (78) linked a decrease in the expression of CYP3A25 with liver inflammation in C57BL6 mice, a model of obesity-dependent diabetes. In our study, only CYP3A62 from the CYP3A family has been decreased after the short- and long-term SPI feeding, whereas CYP3A7 and CYP3A25 have been significantly increased in SPI-fed rats, which can be correlated with the decreased liver inflammation observed in this dietary group. Maximos et al. (78) also related obesity and its detrimental effects to a decrease in the expression of CYP2B and CYP2C family members. A CYP2 impairment is related to a lower drug metabolic clearance (78), mostly CYP2C8 and CYP2C9, both upregulated in our study in the SPI-fed rats. Despite disparities in many human and animal studies, the results show changes (upregulation and downregulation) in CYP expression in an obese and NAFLD rat model after the diet intervention in CYP that have been previously related to obesity and/or the presence of NAFLD.

## Conclusions

In summary, through global gene expression analysis, we found that the gene expression of drug-metabolizing CYP genes was modified in NALFD obese Zucker rat model after being fed a soy-based diet for short and long terms and that this change could have an important role in the attenuation of liver steatosis.

## Data Availability Statement

The datasets presented in this study can be found in online repositories. The names of the repository/repositories and accession number(s) can be found below: https://www.ncbi.nlm.nih.gov/ (GSE158553).

## Ethics Statement

The animal study was reviewed and approved by the Institutional Animal Care and Use Committee at the University of Arkansas for Medical Sciences authorized the animal codes (Protocol code number 3242).

## Author Contributions

RH was involved in experimental design. MK and DA were involved in targeted mRNA analysis. MK, WB, and RH were involved in data analysis and interpretation. MK and DA were involved in writing the original draft preparation. MK, WB, DA, and RH were involved in writing the review and editing. All authors have read and agreed to the published version of this manuscript.

## Conflict of Interest

The authors declare that the research was conducted in the absence of any commercial or financial relationships that could be construed as a potential conflict of interest.
